# Pembrolizumab with or without enzalutamide in selected populations of men with previously untreated metastatic castration-resistant prostate cancer harbouring programmed cell death ligand-1 staining: a retrospective study

**DOI:** 10.1186/s12885-021-08156-1

**Published:** 2021-04-13

**Authors:** Huanyi Lin, Qilong Liu, Xianshang Zeng, Weiguang Yu, Guixing Xu

**Affiliations:** 1 Department of Urinary Surgery, The First Affiliated Hospital, Sun Yat-sen University, No. 58, Zhongshan 2nd Road, Yuexiu District, Guangzhou, 510080 China; 2 Department of Gastrointestinal Surgery, The First Affiliated Hospital, Sun Yat-sen University, No. 58, Zhongshan 2nd Road, Yuexiu District, Guangzhou, 510080 China; 3 Department of Orthopaedics, The First Affiliated Hospital, Sun Yat-sen University, No. 58, Zhongshan 2nd Road, Yuexiu District, Guangzhou, 510080 China; 4 Department of Neurosurgery, The First Affiliated Hospital, Sun Yat-sen University, No. 58, Zhongshan 2nd Road, Yuexiu District, Guangzhou, 510080 China

**Keywords:** Pembrolizumab, Enzalutamide, Castration-resistant, Prostate cancer, Survival

## Abstract

**Background:**

The purpose of this retrospective study was to evaluate the survival outcomes of pembrolizumab (PEM) plus enzalutamide (ENZ) versus PEM alone in selected populations of men with previously untreated metastatic castration-resistant prostate cancer (mCRPC) harbouring programmed cell death ligand-1 (PD-L1) staining.

**Methods:**

Consecutive men with previously untreated mCRPC harbouring PD-L1 staining who underwent treatment with PEM plus ENZ (PE) or PEM alone (PA) at our medical centre from January 1, 2017, to January 31, 2021, were retrospectively identified. Follow-up was conducted monthly during the first year and then every 1 month thereafter. The primary outcomes of the study were overall survival (OS) and progression-free survival (PFS). Secondary outcomes were the frequency of key adverse events (AEs).

**Results:**

In total, 302 men were retrospectively reviewed, 96 of whom were deemed to be ineligible per the exclusion criteria, leaving 206 men (PE: *n* = 100, median age 64 years [range, 43–85] and PA: *n* = 106, 65 years [range, 45–82]) who were eligible for the study. The median follow-up for both groups was 34 months (range, 2–42). At the final follow-up, the median OS was 25.1 months (95% confidence interval [CI], 22.3–27.6) in the PE group versus 18.3 months (95% CI, 16.5–20.9) in the PA group (hazard ratio [HR] 0.56; 95% CI, 0.39–0.80; *p* = 0.001). A marked distinction was also observed in the median PFS (6.1 months [95% CI, 4.7–7.8] for PE vs. 4.9 months for PA (95% CI, 3.2–6.4) for PA; HR 0.55, 95% CI, 0.41–0.75; *p =* 0.001). There were noteworthy differences in the rate of the key AEs between the two groups (72.0% for PE vs. 45.3% for PA, *p* < 0.001). Noteworthy differences were also detected for fatigue events (7.0% in the PE group vs. 0.9% in the PA group, *p* = 0.025) and musculoskeletal events (9.0% for PE vs. 0.9% for PA, *p* = 0.007), but these events tended to be manageable.

**Conclusions:**

Among selected populations of men with previously untreated mCRPC harbouring PD-L1 staining, PEM added to ENZ treatment may significantly increase the survival benefits compared with PEM treatment alone regardless of tumor mutation status. The safety profile for PE plus ENZ tends to be manageable.

## Background

Metastatic castration-resistant prostate cancer (mCRPC) continues to be a major cause of complications and death among men [1–3]. Management of mCRPC is still controversial [3]. Several therapies have been shown to improve the overall survival (OS) and progression-free survival (PFS) of men with mCRPC [4–6]. Nevertheless, the majority of men eventually die as a consequence of mCRPC within 1–3 years. Combining a programmed cell death protein-1 (PD-1) inhibitor (pembrolizumab [PEM]) with a second-generation androgen receptor (AR) antagonist (enzalutamide [ENZ]) at the time of comprehensive therapy for mCRPC has emerged as an option to potentially prolong the progression of cancer and improve OS [7].

**Fig. 1:**
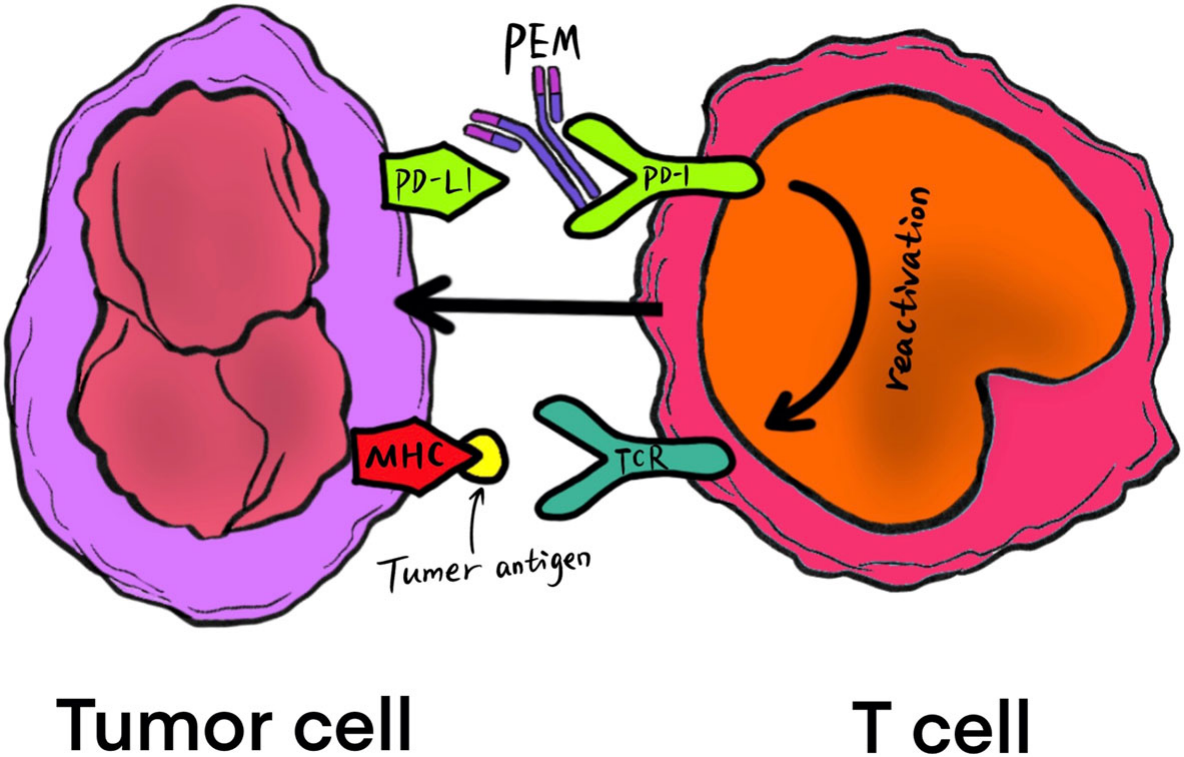
PEM is a humanized monoclonal antibody against PD-1 receptor. The activity of T cells may be restored via the checkpoint blockade using PEM. This binding reactivates the T cell signal induced by the interaction between MHC and TCR by initiating the proliferation of T cells and targeting tumor cells. PEM: pembrolizumab; PD-1: programmed cell death protein-1; MHC: major histocompatibility complex; TCR: T cell receptor

PEM, an IgG4 subclass antibody and a checkpoint inhibitor, binds with high affinity to the cell surface receptor PD-1 [8, 9]. The rationale of an immunological treatment in prostate cancer is shown in Fig. 1. The binding of PEM to PD-1 blocks the inhibitory pathway, triggering a physiological shift to immune reactivity and enhancing the antitumor immune response, and PEM therapy is under development for the management of mCRPC [10, 11]. A recent study [12] showed that one-third of mCRPC biopsies exhibit programmed cell death ligand-1(PD-L1) staining, implying that strategies to enhance CD8+ T cell function and block the inhibitory pathway in the tumor may provide a theoretical basis for the feasibility of mCRPC immunotherapy. Although adding PEM to ENZ treatment has been reported to extend OS for men with mCRPC [13], the activity of PEM monotherapy remains controversial in mCRPC [14]. Immunotherapy with PEM tends to be an ideal treatment option in men with mCRPC, whether as a single agent or in combination with ENZ [9]. Nevertheless, previous pivotal trials [9, 11, 15] with immunotherapy failed to exclude men treated with chemotherapy and/or radiotherapy as well as androgen-deprivation therapy (ADT).

Given the binding of PEM to PD-1, it is unclear whether to stratify PD-1 staining during the final survival analysis, although previous analyses were powered for the entire population and not the subgroups [16, 17]. Furthermore, with previous trials [18, 19] indicating that 2–12% of mCRPC harbour a hypermutated state, PEM epitomizes a different anticancer option for a subset of mCRPC. Nevertheless, remarkably, only a few men with mCRPC were included in the PEM study [13, 20]; hence, the actual efficacy of PEM, even in a biomarker-selected mCRPC setting, has not been fully assessed. Additionally, a retrospective analysis involving select Chinese men with previously untreated mCRPC harbouring PD-L1 staining is lacking. Herein, we performed a retrospective review to verify whether men with previously untreated mCRPC harbouring PD-L1 staining who underwent treatment with PEM plus ENZ had a greater survival advantage than those who underwent treatment with PEM alone.

## Methods

### Data

Consecutive men with previously untreated mCRPC harbouring PD-L1 staining who received PEM plus ENZ (PE) or PEM alone (PA) from January 1, 2017, to January 31, 2021, and for whom patient characteristics were available were retrospectively identified from the First Affiliated Hospital, Sun Yat-sen University. Eligible patients were men with biopsy-proven adenocarcinoma of the prostate with metastatic prostate adenocarcinoma confirmed by sensitive imaging evidence of disease spread (whole-body magnetic resonance imaging [MRI] allowing the early detection of metastases, technetium-99 bone scans, or positron-emission tomography [PET] with prostate-specific membrane antigen [PSMA]); PD-L1 expression staining (combined positive score [CPS] ≥ 1) as determined by an FDA-approved test; PD-L1 expression was measured using PD-L1 IHC 22C3 pharmDx; microsatellite instable-high status; no prior treatment for mCRPC; at least 3 increasing serum prostate-specific antigen (PSA) values based on the Prostate Cancer Working Group guidelines [21]; an Eastern Cooperative Oncology Group (ECOG) performance status from 0 to 1; and adequate heart, liver and renal function [22]. Key exclusion criteria included unavailable pathologic and laboratory data; prior use of checkpoint inhibitor(s); no evaluable CPS; prior chemotherapy and/or radiotherapy, ADT, or surgery for cancer; interruption of PE or PA, regardless of treatment-induced toxicity or adverse events (AEs); active autoimmune disease; neurologic symptoms or symptomatic central nervous system (CNS) metastasis; cachexy; corticosteroid requirement; cauda equina syndrome; some medical illnesses (i.e., pneumonitis, inflammatory bowel disease, uninhibited hypertension, acute respiratory distress syndrome, septicaemia, or septicopyaemia); surgical emergency (i.e., enterobrosis, lienal rupture); major organ failure (i.e., lung, brain, kidney, and/or heart); uncontrolled intercurrent illness; epilepsy; and neuropathy grade 2 or worse.

### Study design and treatment

A retrospective single-centre review was performed in which eligible men had received at least one dose of the PE or PA regimen for the management of mCRPC harbouring PD-L1 staining. The PE regimen consisted of PEM (200 mg intravenously over 30 min every 3 weeks [13] plus ENZ (160 mg/day) [23]. The PA regimen consisted of 200 mg PEM given intravenously over 30 min every 3 weeks. The regimens for PE and PA were terminated with the occurrence of disease progression, toxic effects, or death. Dose modification was not permitted.

### Outcomes and assessments

The co-primary outcomes were OS, defined as the date of drug treatment to death from any cause, and PFS, defined as the date of drug treatment to either disease progression or death from any cause, whichever occurred first. Symptomatic events involving worsening cancer-related symptoms and/or new cancer-related complications were considered clinical progression [24]. Radiologic progression was defined as equal to or greater than a 20% enlargement in sum diameter of soft-tissue target lesions on a computerized tomography (CT) image, equal to or greater than 2 new bone lesions on a bone scan, or death, whichever came first [24]. Disease evaluation was performed every 3 months according to the Response Evaluation Criteria in Solid Tumors (RECIST, v1.1) [25]. PSA50 was defined as PSA decline of ≥50% from baseline PSA level prior to drug initiation. This follow-up schedule continued until disease progression, uncontrollable drug-related toxic effects, or death. Secondary outcomes were the frequency of key drug-related AEs that were evaluated based on the National Cancer Institute Common Terminology Criteria for Adverse Events, v4.0 [26]. Efficacy data for both cohorts were analysed separately. Men with mCRPC were examined centrally for PD-L1 by immunohistochemistry. Follow-up time was defined as the date of drug treatment to the last follow-up. Follow-up was conducted monthly during the first year and then every 3 months thereafter.

### Statistic1al analysis

A χ^2^ test was used to test for differences between categorical data. Continuous data were compared with Student’s t-test for normally distributed data and the Mann-Whitney U test for non-normally distributed data. Survivorship curves were drawn using the Kaplan-Meier method with a log-rank test. The hazard ratio (HR) was calculated using a Cox proportional hazards model, with age used as a covariate and therapy as the time-dependent factor. The median follow-up was estimated using the reverse Kaplan-Meier method. Two-sided 5% level tests were used, without adjustment for multiplicity. The survival curves were generated by GraphPad Prism 8.0(La Jolla, California, USA). We performed the other statistical analyses using SPSS 26.0 (IBM, Inc., NY, America).

## Results

### Demographic characteristics

**Fig. 2:**
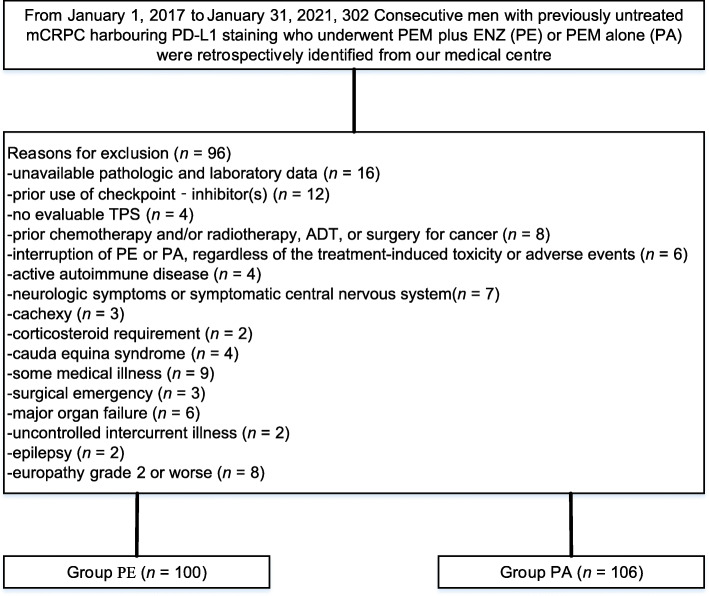
Flow diagram exhibiting the methods applied to identify objects to evaluate survival of pembrolizumab plus enzalutamide (PE) versus pembrolizumab alone (PA) in selected men with previously untreated metastatic castration-resistant prostate cancer harbouring programmed cell death ligand-1 staining

We identified 302 men with previously untreated mCRPC harbouring PD-L1 staining, of whom 206 were included. Of these men, 100 men underwent PE, and 106 underwent PA, as shown in Fig. 2. Table 1 summarizes the baseline data of men who underwent PE or PA. Baseline data were well balanced between groups. The median age was 64 years (range, 43–85) in the PE group and 65 years (range, 45–82) in the PA group. The median time since diagnosis for both groups was 7 months. PD-L1 CPS was 1–20 in 64.0%, 20–50 in 22.0%, and 50–100 in 14.0% of men undergoing PE versus 1–20 in 63.2%, 20–50 in 21.7%, and 50–100 in 15.1% of men undergoing PA (*p* = 0.872). Asymptomatic CNS metastasis and no CNS metastasis were 35.0 and 65.0% in the PE group versus 45.3 and 54.7% in the PA group, respectively (*p* = 0.134). ECOG status was 0 in 38.0% and 1 in 62.0% of men undergoing PE versus 0 in 41.5% and 1 in 58.5% of men undergoing PA (*p* = 0.608). Additionally, no noteworthy distinctions were observed in terms of age, PSA level, alkaline phosphatase level, albumin, clinical stage at diagnosis, pathological stage at diagnosis, Gleason sum at diagnosis, or the number of metastatic sites. The median follow-up for the study was 34 months (range, 2–42). The median number of treatment cycles was 33 (range, 1–42) for men receiving PE and 34 (range, 1–42) for those who were treated with PA.

**Table 1:** Characteristics of patients who underwent treatment

Variable	PE (*n* = 100)	PA (*n =* 106)	*p*-value
Age, years	64 (43–85)	65 (45–82)	0.227^a^
Time since diagnosis, month(s)	7 (2–15)	7 (1–17)	0.361^a^
PSA level^c^, ng/mL	121 (2–2165)	126 (4–2110)	0.183^a^
Alkaline phosphatase level^c^, U/L	181 (72–1023)	173 (66–983)	0.219^a^
Albumin g/dl	3.6 (2.2–3.9)	3.7 (2.1–4.2)	0.127^a^
Clinical stage at diagnosis, n (%)			0.794^b^
T1c	9 (9.0)	7 (6.6)	
T2b	12 (12.0)	14 (13.2)	
T2c	14 (14.0)	20 (18.8)	
T3	20 (20.0)	21 (19.8)	
M0	22 (22.0)	20 (18.8)	
M1	23 (23.0)	24 (22.6)	
Pathological stage at diagnosis, n (%)			0.297^b^
T2	22 (22.0)	20 (18.9)	
T3	28 (28.0)	26 (24.5)	
N0	30 (30.0)	33 (31.1)	
N1	20 (20.0)	27 (25.5)	
Gleason sum at diagnosis, n (%)			0.941^b^
≤ 6	23 (23.0)	24 (22.6)	
7	35 (35.0)	37 (34.9)	
≥ 8	42 (42.0)	45 (42.4)	
PD-L1 expression (CPS cut-off values), n (%)			0.872^b^
1–20	64 (64.0)	67 (63.2)	
20–50	22 (22.0)	23 (21.7)	
50–100	14 (14.0)	16 (15.1)	
mCRPC-related brain metastasis, n (%)			0.134^b^
Asymptomatic CNS metastasis	35 (35.0)	48 (45.3)	
Without CNS metastasis	65 (65.0)	58 (54.7)	
Duration of drug treatment (months)	16 (1–34)	17 (2–33)	0.103^a^
ECOG status, n (%)			0.608^b^
0^d^	38 (38.0)	44 (41.5)	
1^e^	62 (62.0)	62 (58.5)	
No. of metastatic sites, n (%)			0.597^b^
3	23 (23.0)	31 (29.2)	
> 3	62 (62.0)	57 (53.8)	
Unclear	15 (15.0)	18 (17.0)	

^a^*Independent samples t-test;* ^b^ *Mann-Whitney U test;* ^c^ *Calculated one week prior to the start of PE or PA; 0*^d^*, Fully active, able to carry on all pre-disease performance without restriction; 1*^e^*, Restricted in physically strenuous activity but ambulatory and able to carry out work of a light or sedentary nature,* i.e.*, light housework, office work*

*PE* pembrolizumab plus enzalutamide*, PA* pembrolizumab alone, *PSA* prostate-specific antigen, *CNS* central nervous system, *PD-L1* programmed cell death ligand-1, *CPS* combined positive score, *mCRPC* metastatic castration-resistant prostate cancer, *ECOG* Eastern Collaborative Oncology Group

**Fig. 3:**
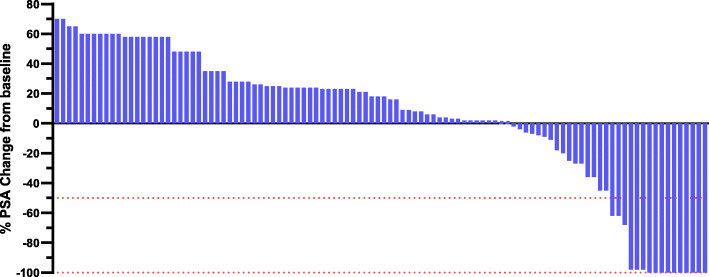
Best PSA change from baseline in PA-treated men with previously untreated metastatic castration-resistant prostate cancer harbouring programmed cell death ligand-1 staining who underwent pembrolizumab alone (*n =* 106)

**Fig. 4:**
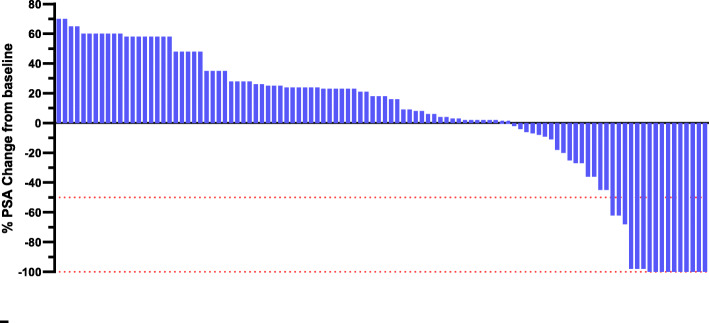
Best PSA change from baseline in PE-treated men with previously untreated metastatic castration-resistant prostate cancer harbouring programmed cell death ligand-1 staining who underwent pembrolizumab alone (*n =* 100)

Fourteen (13.2%) PA-treated men received ENZ prior to death. Figure 3 showed that the best PSA change from baseline in PA-treat men with previously untreated mCRPC harbouring PD-L1 staining. Sixteen men (15.1%) achieved PSA50 after a median of 2 months (range, 1–5) following treatment initiation. Among 16 men, thirteen men had more than 97% PSA decline, and ten men had 100% PSA decline. Figure 4 showed that the best PSA change from baseline in PE-treat men with previously untreated mCRPC harbouring PD-L1 staining. Nineteen men (19.0%) achieved PSA50 after a median of 2 months (range, 1–4) following treatment initiation. Among 19 men, 12 men had 100% PSA decline. The overall response rate was 66% in the PE group versus 56% in the PA group (*p* < 0.05).

### Survival analysis

**Fig. 5:**
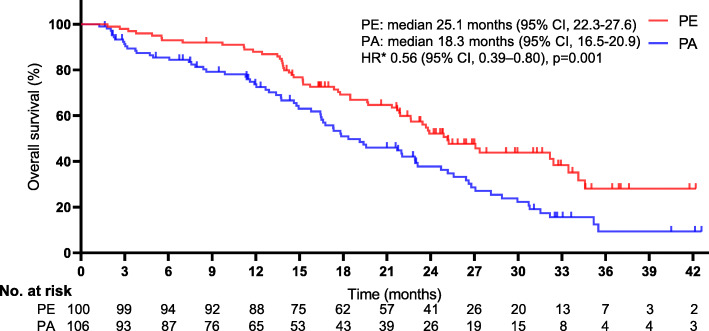
Kaplan-Meier curves for overall survival. The median overall survival was 25.1 months (95% confidence interval [CI], 22.3–27.6) for PE and 18.3 months (95% CI, 16.5–20.9) for PA (HR 0.56, 95%CI, 0.39–0.80; *p* = 0.001). *The hazard ratio was calculated using a Cox proportional hazards model, with the age, time since diagnosis, PSA level, alkaline phosphatase level, clinical stage at diagnosis, pathological stage at diagnosis, Gleason sum at diagnosis, mCRPC-related brain metastasis, ECOG status, and number of metastatic sites used as covariates and therapy as the time-dependent factor

**Fig. 6:**
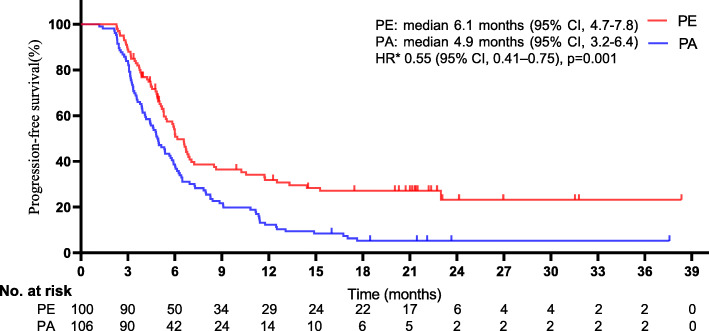
Kaplan-Meier curves for progression-free survival. The median progression-free survival was 6.1 months (95% confidence interval [CI], 4.7–7.8) for PE and 4.9 months (95% CI 3.2–6.4) for PA (HR 0.55, 95%CI, 0.41–0.75; *p =* 0.001). *The hazard ratio was calculated using a Cox proportional hazards model, with the age, time since diagnosis, PSA level, alkaline phosphatase level, clinical stage at diagnosis, pathological stage at diagnosis, Gleason sum at diagnosis, mCRPC-related brain metastasis, ECOG status, and number of metastatic sites used as covariates and therapy as the time-dependent factor

Figures 5 and 6 show the Kaplan-Meier survival curves for the distinctions between the groups in OS and PFS, respectively. At the end of the follow-up, 125 deaths were registered (60.7% [125/206]; 54 men [54%] in the PE group vs. 71 men [67.0%] in the PA group). In the PE group, 38 deaths (38%) were mCRPC-related, and 16 (16%) were not mCRPC-related; in the PA group, 62 deaths (58.5%) were CRPC-related, and 9 (8.5%) were not. The 54 men who died in the PE group had a median age of 78 years (range, 52 to 85), and the 71 men who died in the PA group had a median age of 79 years (range, 55–82). A borderline remarkable difference was found in the median OS between groups (25.1 months [95% CI, 22.3–27.6] for PE vs. 18.3 months [95% CI, 16.5–20.9] for PA). The addition of ENZ to PEM treatment notably improved the median OS versus treatment with PEM alone, and PEM plus ENZ was associated with a noteworthy 44% lower risk of death than PEM alone (HR 0.56, 95% CI, 0.39–0.80; *p* = 0.001). A remarkable difference of approximately 7 months was detected in the median OS, and the advantage of PE over PA was significant, as the two curves continued to separate until the end of follow-up. Additionally, a marked distinction in the median PFS between groups was noted (6.1 months [95% CI, 4.7–7.8] for PE vs. 4.9 months for PA [95% CI, 3.2–6.4] [HR 0.55, 95% CI, 0.41–0.75; *p* = 0.001]). Of the 206 men, PE or PA was terminated in 54 (26.2%), mostly due to disease progression (34 of 54 men in the PE group vs. 20 of 54 men in the PA group, *p* = 0.014). Although more men eventually developed disease progression in the PE group, a noteworthy delay was seen in the time taken for tumors to progress in this group, which may have contributed to the appreciably longer PFS.

In a subset analysis based on PDL1 expression, the median OS in the PE versus PA group was 28.6 months vs. 21.3 months among men with PD-L1 CPS ≥50 (HR 0.62; *p* = 0.001), 26.6 months vs. 19.4 months among those with a CPS ≥20 (HR 0.43; *p =* 0.001) and 21.4 months vs. 16.8 months among those with a CPS ≥1 (HR 0.53, *p =* 0.001). The higher the level of tumor PD-L1 expression indicating the presence of an antitumor immune response, the more likely it will be that the individual will benefit from PE therapy regardless of tumor mutation status.

### Adverse events

The incidence of the key AEs during the first three follow-ups was higher in the PE group than in the PA group. Throughout the follow-up period, cardiovascular events were serious AEs resulting in death. There was no significant difference in cardiovascular events (2 men [1.0%] in the PE group vs. 2 [1.9%] in the PA group, *p =* 0.953). The most frequently reported AEs were primarily immune-related events, hypothyroidism, hyperthyroidism, fatigue, and musculoskeletal events. Table 2 shows the frequency of the key AEs possibly associated with PE or PA. In the PE group, the frequency of the key AEs was 72.0% (72 AEs in 65 men). In the PA group, the frequency of the key AEs was 45.3% (48 AEs in 55 men). PE-treated men had a higher rate of key AEs than PA-treated men (72.0% vs. 45.3%, *p* < 0.001). Noteworthy distinctions were observed for fatigue events (7.0% in the PE group vs. 0.9% in the PA group, *p* = 0.025) and musculoskeletal events (9.0% in the PE group vs. 0.9% in the PA group, *p* = 0.007), but these events tended to be manageable. There were no noteworthy distinctions between the two cohorts in terms of immune-related events, hypothyroidism, hyperthyroidism, thyroiditis, pneumonitis or interstitial lung disease, vitiligo, colitis, fracture, hypertension, falls, cognitive and memory impairment, or cardiovascular events.

**Table 2:** Key adverse events

Variable, n%	PE (*n =* 100)	PA (*n =* 106)	HR (95%)	*p-*value
Immune-related event	21 (21%)	16 (15.1)	2.00 (0.21–3.53)	0.271
Hypothyroidism	11 (11.0)	8 (7.5)	4.00 (0.38–2.71)	0.393
Hyperthyroidism	7 (7.0)	5 (4.7)	1.00 (0.12–3.43)	0.485
Thyroiditis	3 (3.0)	2 (1.9)	2.00 (0.37–4.81)	0.605
Pneumonitis or interstitial lung disease	3 (3.0)	2 (1.9)	2.00 (0.74–4.35)	0.605
Vitiligo	1 (1.0)	1 (0.9)	4.00 (0.28–6.54)	1.000
Colitis	2 (2.0)	2 (1.9)	3.00 (0.43–4.22)	0.953
Fatigue (asthenia)	7 (7.0)	1 (0.9)	3.00 (1.79–3.88)	0.025^a^
Musculoskeletal event^b^	9 (9.0)	1 (0.9)	5.00 (3.65–6.85)	0.007^a^
Fracture^c^	1 (1.0)	4 (3.8)	3.00 (0.31–4.76)	0.196
Hypertension^d^	1 (1.0)	2 (1.9)	2.00 (0.28–3.41)	1.000
Fall	2 (2.0)	1 (0.9)	2.00 (0.83–3.73)	0.612
Cognitive and memory impairment^e^	2 (2.0)	1 (0.9)	2.00 (0.14–3.47)	0.612
Cardiovascular event	2 (1.0)	2 (1.9)	3.00 (0.27–4.19)	0.953

^a^*Statistically significant*

^b^*included musculoskeletal pain, pain in extremity, arthralgia, myalgia, stiffness, muscular weakness, and muscle spasms;* ^c^ *included bone and joint injuries;* ^d^ *included hypertensive retinopathy, increased blood pressure, systolic hypertension, and hypertensive crisis;* ^e^ *included cognitive disorders, amnesia, Alzheimer’s disease, dementia, senile dementia, mental impairment, and vascular dementia*

*PE* pembrolizumab plus enzalutamide, *PA* pembrolizumab alone, *HR* Hazard ratio

## Discussion

The findings from the current retrospective review demonstrated that the addition of PEM to ENZ treatment may be associated with substantially longer PFS times for men with previously untreated mCRPC harbouring PD-L1 staining, prominently longer OS than the use of PEM treatment alone, and a manageable safety profile. The Kaplan-Meier curves for survival among the men in our study suggested an early survival advantage for men undergoing treatment with PEM plus ENZ that continued until the last follow-up, with a remarkable difference of approximately 7 months in median OS, which reached statistical significance regardless of tumor mutation status.

The results of this retrospective review are consistent with the findings from a phase II single-arm study of 28 men with mCRPC [13], which examined the antitumor effect of PEM added to ENZ treatment in men with mCRPC whose cancer was progressing with ENZ treatment alone. In that study, the median OS for all men was 21.9 months (95% CI, 14.7 to 28.4 months). Their conclusion showed that the PEM plus ENZ regimen is effective in mCRPC, and responses did not require tumor PD-L1 expression. Recently, a multicohort, open-label phase II KEYNOTE-199 study [27] of 258 men with mCRPC assessed the antitumor activity and safety of PEM in three parallel cohorts who had undergone treatment with docetaxel and one or more targeted endocrine therapies. All men received 200 mg PEM every 3 weeks for up to 35 cycles. The study showed that the objective response rate was 5% (95% CI, 2 to 11%) for RECIST-measurable PD-L1-positive men and 3% (95% CI, < 1 to 11%) for RECIST-measurable PD-L1-negative men. The median OS was 9.5 months for RECIST-measurable PD-L1-positive men, 7.9 months for RECIST-measurable PD-L1-negative men, and 14.1 months for men with bone-predominant disease, regardless of PD-L1 expression. Although the mechanisms of response and resistance to docetaxel and ≥ 1 targeted endocrine therapy are still unclear, an explanation as to why a higher median OS (25.1 months) was observed in the present study might be that the earlier the PEM is used, the greater the survival benefit for the men. In previously treated mCRPC, some men have a therapeutic response to PEM [13]. Those who do respond to anti-PD-1/PD-L1 therapy have a distinct, durable response to treatment, suggesting some derive long-term benefit from PEM [27, 28]. The effect of earlier versus later treatment with PEM may have already been felt. Previous studies [13, 27] have found a diminished survival benefit attributable to the lack of effective therapies for mCRPC following the development of resistance to traditional remedies. Furthermore, distinct dissimilarities are observed in the duration of PEM treatment utilised in these studies for men with an initial diagnosis of mCRPC. Variation in PEM treatment duration may contribute to the differences in survival outcomes.

PEM, a highly selective IgG4-kappa humanized monoclonal antibody against the PD-1 receptor, has shown a high response rate in mCRPC with mismatch repair deficiency, regardless of the primary tumor site [29], and was first approved by the FDA on September 4, 2014. The expression of PD-1 is associated with the aggressiveness of mCRPC and results in poor survival [27, 30]. A previous study [11] of 54 consecutive men with progressive, recurrent, or advanced prostate cancer who were treated with 1 to 12 cycles of 200 mg PEM every 3 weeks demonstrated that PEM had modest anticancer activity against mCRPC.

Although the efficacy of PEM plus ENZ treatment in prolonging survival is promising, definite differences are observed in the duration of PEM treatment utilized in prior reports [14, 20] for men with an initial diagnosis of mCRPC. PEM is provided until castration resistance and/or disease progression, which may lead to differences in conclusions [27]. The delivery of PEM and ENZ is not synchronized in time, and this may have an effect on clinical decision making [13]. Comparisons of the PEM plus ENZ regimen in the present study with the PEM-based regimen in previous studies in accordance with the duration of therapy have not yet been implemented. Data from studies establishing whether the superiority of contemporaneous administration of PEM and ENZ instead of serial administration need to be explored further.

Prostate cancer has inherent characteristics [31]. Its growth and development are highly dependent on androgens [32]. ADT is an effective strategy and is widely used in clinical practice [33]. For men with asymptomatic or symptomatic mCRPC, first-line therapies usually involve ENZ (a targeted AR inhibitor) [31]. ENZ has the ability to bind to the ligand-binding domain of AR and to hinder the translocation of AR to the nucleus and the binding of AR to DNA [34]. Findings from an extended analysis of the phase 3 PREVAIL study [23] assessing ENZ treatment in men with chemotherapy-naive mCRPC showed that the median OS was 35.3 months (95% CI, 32.2-not reached) in the ENZ arm and 31.3 months (95% CI, 28.8–34.2) in the placebo arm. Moreover, the survival benefit conferred by the addition of ENZ to ADT tends to be sustained with extended follow-up, as evidenced by sustained separation of the OS curves. The reported AFFIRM trial [35] in 1199 men with previous docetaxel exposure (2,1 to ENZ or placebo) showed that a significant difference was detected in the median OS (18.4 months for ENZ vs. 13.6 months for placebo).

Several key shortcomings should be acknowledged. First, the present retrospective review had inherent weaknesses. Residual confounding variables may lead to overestimation of the survival curve. Second, the identification of disease metastasis was not performed with PSMA PET in all men, and inaccurate imaging may convert the initial diagnosis from metastatic to nonmetastatic disease, eventually leading to the overestimation of the conclusions of the study. Third, the present subjects included were limited to men with previously untreated mCRPC harbouring PD-L1 staining; therefore, generalizability for men with mCRPC is lacking in this study. Fourth, although the AR splice variant 7 may serve as a treatment selection marker [24], detection of the AR splice variant 7 was not performed.

## Conclusion

The results described here support the increasing body of evidence indicating that the addition of PEM treatment to ENZ therapy is associated with increased survival benefits in men with previously untreated mCRPC harbouring PD-L1 staining regardless of tumor mutation status. Additionally, the clinical outcomes of long-term maintenance therapy with PEM plus ENZ need further exploration in these men.

## Data Availability

The datasets generated during and/or analysed during the current study are not publicly available due to privacy regulations but are available from the corresponding author on reasonable request.

## Abbreviations

PEM: Pembrolizumab
ENZ: Enzalutamide
mCRPC: Metastatic castration-resistant prostate cancer
PD-1: Programmed cell death protein-1
PD-L1: Programmed cell death ligand-1
MHC: Major histocompatibility complex
TCR: T cell receptor
PE: PEM plus ENZ
PA: PEM alone
OS: Overall survival
PFS: Progression-free survival
AEs: Adverse events
CI: Confidence interval
HR: Hazard ratio
ADT: Androgen-deprivation therapy
MRI: Magnetic resonance imaging
PSMA: Prostate-specific membrane antigen
CPS: Combined positive score
ECOG: Eastern Collaborative Oncology Group
CNS: Central nervous system
CT: Computed tomography
BMI: Body mass index
RECIST: Response Evaluation Criteria in Solid Tumors
